# Obsession to fairness and topical steroid induced acne: A situation analysis of patients presenting in dermatology clinic at a private hospital in Karachi

**DOI:** 10.12669/pjms.40.1.7074

**Published:** 2024

**Authors:** Afza Naureen Ghouse, Erum Ashraf, Zara Iqbal Khan, Sitwat Sidiqqui

**Affiliations:** 1 Afza Naureen Ghouse Consultant Dermatologist, Indus Hospital, Karachi, Pakistan; 2 Erum Ashraf Consultant Dermatologist, Institute of Skin Diseases Karachi, Pakistan; 3 Zara Iqbal Khan Consultant Dermatologist, Glamor Medical Centre Riyadh, Saudi Arabia; 4 Sitwat Sidiqqui Consultant Dermatologist, Patel Hospital, Karachi, Pakistan

**Keywords:** Acne, Topical Corticosteroids, Fairness Cream Use

## Abstract

**Objective:**

To determine the frequency of acne and other relevant side effects as well as the pattern of topical steroid and fairness cream use among patients presenting with steroid and fairness cream use at dermatology OPD in a tertiary care private hospital in Karachi.

**Methods:**

A cross-sectional survey was conducted from April, 2020 to December, 2020 in a private tertiary care hospital in Karachi. In total, 226 patients with a positive history of topical steroids and/or fairness creams use in the past six months were included in the study. Information was collected about sociodemographic characteristics; topical corticosteroid uses while clinical examination of facial skin was performed by a dermatologist. Data were analyzed using SPSS version-19.

**Results:**

The median age of study participants was 26 years with an interquartile range of 10 years. This frequency of corticosteroid induced acne was highest i.e., 83.6% (n=189) followed facial erythema and telangiectasia i.e., 50.9% (n=115) 47.8% (n=108) respectively. The estimated median duration of using topical steroids or fairness creams or both was six months with an IQR of four months. The study found statistically significant differences in the reasons of using topical corticosteroids or fairness creams on the face on the basis of differences in the level of education and marital status.

**Conclusion:**

In Karachi, both, men and women are equally obsessed with fair skin tone and use topical steroids and fairness cream. The use of corticosteroid or fairness cream-induced facial acne is alarmingly high among patients presenting in a dermatology clinic in Karachi.

## INTRODUCTION

Globally, fair skin color is considered a prime beauty standard including East Asia, South Asia, Middle East, and African regions.^1^,^2^ The major reason behind this obsession is increasing social and psychological pressures to meet beauty standards i.e. fair skin tone as defined under the influence of culture and media.^2^,^3^ This worldwide obsession for fair skin has caused an alarming increase in the irrational use of topical corticosteroids and fairness creams on the face.^1^-^3^ Studies conducted in India, Nepal, Saudi Arabia, and Ethiopia have reported a considerable burden of irrational use of topical corticosteroids and fairness creams to lighten the skin tone.^4^-^6^

The irrational use of topical corticosteroids and fairness creams may lead to many serious adverse effects on the skin including acne, skin atrophy, striae, rosacea, perioral dermatitis, acne, and purpura.7-9 Hence, the adverse effects of topical corticosteroids and fairness creams make their wide use a huge public health threat for the exposed populations. Pakistan is a country where social and cultural factors play a crucial role in the preference of fair skin complexion.^10^-^12^ Hence, this study aimed to determine the frequency of acne and other relevant side effects as well as the pattern of topical steroid and fairness cream use among patients presenting with steroid and fairness cream use at dermatology OPD in a tertiary care private hospital in Karachi. Understanding the current situation of acne and other cutaneous manifestations among patients exposed to topical corticosteroid use and fairness creams on the face will help generate relevant evidence for implementing appropriate control and prevention measures.

## METHODS

A cross-sectional survey was conducted from April, 2020 to December, 2020 in a private tertiary care hospital in Karachi. Patients of age between 10 years to 40 years, visiting Dermatology Out-Patient Department (OPD) with history of topical corticosteroids and/or fairness creams on the face in past six months, continuously for one month and/or intermittently for more than three months.

### Exclusion Criteria

However, any patient using systematic corticosteroids for any skin disease or any other medical problem was excluded from the study. Similarly patients with preexisting acne vulgaris or acne due to other causes or using systematic corticosteroids for any skin disease or other medical problem were excluded from the study. Moreover, patients having endocrine disorders or females with polycystic ovary syndrome were also excluded from the study.

Non-probability convenient sampling was applied to select the study participants from dermatology OPD. The sample size for this study was calculated using Open Epi sample size calculator and obtained a sample size of 226 with an anticipated population proportion of 15% for topical steroid use.^13^,^14^

The Data was collected using a structured questionnaire which was translated into the local language i.e., “Urdu”. Information was collected regarding, basic socio-demographic characteristics, presence of acne on the face and conditions related to abuse of topical corticosteroids was collected by detailed clinical examination and the use of topical corticosteroids or fairness creams.

### Ethical Approval

The ethical approval for this study was conducted by the Ethics Review Committee of Patel Hospital Karachi with registration: No.: 79 on February 28^th^ 2020.

### Data Analysis

Data was analyzed using SPSS version-19. Descriptive statistics were calculated for socio-demographic characteristics. Chi-square test was used to assess any statistically significant differences in the frequency of acne, cutaneous side effects, and use of topical corticosteroid or fairness cream. P-value of 0.05 or less was considered statistically significant.

## RESULTS

A sample of 226 study participants was collected. The median age of study participants was 26 years with an interquartile range of 10 years (Table-I).83.6% (n=189) of the study participants were found to have acne while 16.4% (n =37) were found to have no acne on physical examination by the dermatologist. Papules were the most frequent inflammatory lesion with a proportion of 82.4% followed by pustules, nodules, and cysts. Open comedones were the most common non-inflammatory lesions with followed by closed comedones and black and white heads with the proportion of 55.9 % (n=33), 37.2% (n=22) and 6.7% (n=7) respectively.

**Table-I:** Socio-demographic characteristics, pattern of topical costicosteroids or fairness cream use on face and related health characteristics of skin patients with history of topical corticosteroids.

Variable	Frequency (n)	Percentage (%)
***Median Age 26 years (IQR =10 years)***		
***Age (in completed years)***		
18 years or younger	43	19.5
19-25 years	69	30.0
26 years and above	114	50.5
***Sex***		
Male	46	20.4
Female	180	79.6
***Marital status***		
Single	138	61.1
Married	82	36.3
Divorced	6	2.7
***Education***		
Primary or less	25	11.1
Middle- Secondary	63	27.9
Intermediate and above	138	61.1
***Monthly household income***		
20,000 PKR or less	32	14.2
210000-50,000	129	57.1
More than 50,000	65	28.8
***Median duration of using topical steroid or fairness creams***		
***6 months (IQR= 4 months)***		
***Pattern of using fairness cream or steroid***		
Use fairness cream	129	57.1
Use Steroid	68	30.1
Use both (fairness cream and steroid)	29	12.8
***Reason for using topical steroids***		
For Fairness	165	73.0
For any reason other than fairness	61	27.0
***Use of topical steroids or fairness cream advised by***		
Doctor	23	10.3
Pharmacist	31	13.7
Family member/ Friends/Neighbours	103	45.6
Beautician	10	4.4
Other	59	26.1
***Reason for current visit to dermatologist***		
Acne	116	51.3
Melasma	32	14.2
Acne with Eczema or Melasma	26	11.5
For fairness treatment	6	2.7
Fungal infection	15	6.6
Others *	31	13.7
Side-effects other than acne as identified on clinical examination**		
Skin Atrophy	50	22.1
Facial Erythema	107	47.3
Telangiectasia	115	50.9
Hypertrichosis	108	47.8
Rosacea	9	4.0
Hypopigmentation	10	4.4
Aggravation of existing dermatosis	13	5.8
***Type of acne identified on examination***		
No Acne	37	16.4
Inflammatory lesions	130	57.5
Non-inflammatory lesions	07	03.1
Inflammatory as well non-inflammatory lesions	52	23.0

* Include photosensitivity, alopecia, sunburn, Herpes stomatitis and Lichen planus,

** Multiple responses possible.

Among all the study participants 30.1% (n = 68) of the participants reported use of topical corticosteroids only, 57.1% (n=129) reported using fairness cream while 12.8% of the study participants were using both i.e., topical corticosteroids as well as fairness cream on their face (Table-I).

This study observed a higher frequency of acne among patients of age 18 years or less as compared to other age groups and among patients who were single as compared to married and divorced. Both these findings were statistically significant (p-value ≤0.05) (Table-II). This study found statistically significant differences in the reasons for using topical corticosteroids and fairness creams on the face on the basis of different socio-demographic characteristics such as level of education and marital status (Table-III).

**Table-II:** Frequency distribution of acne among patients with history of steroids and fairness cream use on the basis of basic socio-demographic characteristics and pattern of use (n=226).

Variable	Acne	No acne	p-value
***Age (in completed years)***			0.05
18 years or younger	40(93.0)	3(7.0)	
19-25 years	60(87.0)	9(13.0)	
26 years and above	89(78.1)	25(21.9)	
***Sex***			0.27
Male	36(78.3)	10(21.7)	
Female	153(85.0)	27(15.0)	
Marital status*			0.001*
Single	125(90.6)	13(9.4)	
Married	58(70.7)	24(29.3)	
Divorced	0	6(100.0)	
***Education***			0.46*
Primary or less	23(92.0)	2(80.0)	
Middle-Secondary	51(81.0)	12(19.0)	
Intermediate and above	115(83.3)	23(16.7)	
***Monthly household income***			0.36
20,000 PKR or less	24(75.0)	8(25.0)	
210000-50,000	111(86.0)	18(14.0)	
More than 50,000	54(83.1)	11(16.9)	
***Pattern of using fairness cream or steroid***			0.87*
Use fairness cream	109(84.5)	20(15.5)	
Use Steroid	24(82.8)	5(17.2)	
Use both (fairness cream and steroid)	56(82.4)	12(17.6)	
***Purpose of use***			0.68
For fairness	139(84.2)	26(15.8)	
For any other reason	50(82.0)	11(18.0)	

**Table-III:** Frequency distribution of steroid or fairness cream use purpose in context of basic socio-demographic characteristics of the study participants (n=226).

Variable	For Fairness	For Other Reasons	p-value
***Age (in completed years)***			
18 years or younger	31(72.1)	12(27.9)	0.014
19-25 years	59(85.5)	10(14.5)	
26 years and above	75(65.8)	39(34.2)	
***Sex***			
Male	35(76.1)	11(23.9)	0.71
Female	130(72.2)	50(27.8)	
Marital status*			
Single	109(79.0)	29(21.0)	0.024
Married	53(64.6)	29(35.4)	
Divorced	03(50.0)	3(50.0)	
***Education***			
Primary or less	11(44.0)	14(56.0)	0.002
Middle-Secondary	47(74.6)	16 (25.0)	
Intermediate and above	107(77.5)	31(22.5)	
***Monthly household income***			
20,000 PKR or less	24(75.0)	08(25.0)	0.95
210000-50,000	94(72.9)	35(27.1)	
More than 50,000	47(72.3)	18(27.7)	

* Fisher exact test.

## DISCUSSION

This study is among the few studies conducted in Pakistan estimating the burden of an important and emerging health issue i.e., acne and other cutaneous side effects of using topical steroids and fairness creams. Moreover, this study also assessed the pattern of topical steroid and fairness creams use among patients presenting in a well-known private tertiary care hospital in Karachi. Hence this study provides much needed evidence about the irrational use of topical corticosteroids and fairness creams in a megacity with relatively better literacy rates.

This study found a very high burden of inflammatory and non-inflammatory acne lesions among the study participants i.e. 83.6% Moreover, in this study acne was identified as the most common side-effect of using topical corticosteroid or fairness cream as reported by previous studies.^13^,^15^-^17^ However, the burden of acne in our study population is slightly higher than the frequency reported by previous studies which can be explained by the differences in research methodology. In this study, the burden of acne was significantly higher among study participants of the age group 18 years or less.

The relatively higher burden of acne among the youngest age group in this study can be explained by the increased tendency and sensitivity of teens towards their outlooks leading to excessive use of topical agents as well as puberty-related bodily changes. Literature from previous studies conducted in Pakistan also support the increased obsession for fairness among Pakistani youth and the role of local media in boosting this obsession for fairer skin.^18^,^19^ Among other cutaneous side-effects identified in this study telangiectasia, hypertrichosis and facial erythema were the most frequent observations. This finding is also in line with previous evidence from the studies conducted in Pakistan and other populations with similar socio-cultural contexts.^11^,^13^-^14^,^18^

This study found a relatively higher frequency of fairness cream use followed by the use of topical corticosteroids alone. This finding is in contrast to previous studies conducted in similar populations that can be explained by the cultural obsession for fair skin as well as the strong role of media in promoting fairness products in Pakistan.^11^,^18^,^20^ Furthermore, this study couldn`t find any statistically significant differences in the use of topical corticosteroids and fairness cream use among men and women. Similarly, the desire for fairness was the main reason for using corticosteroids across three household income categories. The increased desire for fair skin among men is supported by previous studies and can be explained by the strong marketing tactics of fairness product manufacturers.^21^,^22^

However, this study found statistically significant differences in the frequency of using topical corticosteroid products for fairness based on age, education, and marital status. The study found that people of age between 20-25 years, people with higher education levels, and those who were single mostly reported a desire for fair skin as the prime reason for using topical corticosteroids and fairness products. These findings are well supported by the socio-cultural environment of Pakistan and other South Asian countries where skin color plays a crucial role in social acceptance and prospects, particularly for women.^1^,^2^,^11^ The demand for fair skin has turned into an obsession because of social influence of media and endorsement of such advertisements by celebrities.^23^,^24^

This study identified that family, friends, or neighbors as the most common source for recommendation or advice regarding the use of topical corticosteroids or fairness cream. This finding is in line with previous studies conducted in Pakistan as well as the Indian population emphasizing the role of social normal and peer pressure to gain a fairer skin due to specific socio-cultural backgrounds.^12^,^23^-^27^

### Limitations

First, this study included participants from one particular private tertiary care hospital in Karachi and lacks the capacity to measure actual prevalence of topical steroid and fairness cream use in Karachi. Secondly, this study ascertained the burden of topical steroid and fairness cream induced acne and other cutaneous side effects. Moreover, the patients presenting in public sector hospitals and those going to aesthetic treatment centers (usually managed by non-medics) might be different which further limits the generalizability of study findings.

Moreover, this study did not collect objective information regarding the exact composition or ingredients, amount or dose, and frequency of topical corticosteroids or fairness creams use for the total reported duration. The subjective recall for a total duration of topical steroid or fairness cream use might be affected by the severity of related medical complications or related health outcomes. However, despite a few limitations this study provides valuable insights to develop and implement targeted control and prevention strategies for this particular population group.

## CONCLUSION

The young population in Karachi is obsessed with fair skin tone resulting in irrational use of topical corticosteroids and fairness cream without any expert advice. This irrational use of topical corticosteroids is equally prevalent in men and women irrespective of average household income however, it is more common among single or unmarried or individuals with education level of intermediate or above. Developing population-level key interventions is crucial while taking into account the socio-cultural and behavioral aspects of this problem.

### Author`s Contribution

**ANG:** Conceived the idea, designed the study, analyzed data and did final edits on the manuscript. She is also responsible for the integrity and accuracy of the study.

**EA:** Participated in analysis and prepared the first draft of the manuscript.

**ZIK:** did the data analysis prepared the concept in the manual and edited the first and second draft.

**SS:** Participated in statistical analysis and wrote methodology section of the manuscript.
